# Preliminary diagnostic criteria for distinguishing metaplastic Warthin tumors from mucoepidermoid carcinomas rich in lymphoid stroma

**DOI:** 10.31744/einstein_journal/2026AO1591

**Published:** 2026-04-07

**Authors:** João Felipe Leite Bonfitto, João Figueira Scarini, Iara Vieira Ferreira, Reydson Alcides de Lima-Souza, Erika Said Abu Egal, Albina Altemani, Fernanda Viviane Mariano

**Affiliations:** 1 Universidade Estadual de Campinas Faculdade de Ciências Médicas Pathology Department Campinas SP Brazil Pathology Department, Faculdade de Ciências Médicas, Universidade Estadual de Campinas, Campinas, SP, Brazil.; 2 Universidade Estadual de Campinas Faculdade de Odontologia de Piracicaba Oral Diagnosis Department Piracicaba SP Brazil Oral Diagnosis Department, Faculdade de Odontologia de Piracicaba, Universidade Estadual de Campinas, Piracicaba, SP, Brazil.; 3 University of Utah Huntsman Cancer Institute Biorepository and Molecular Pathology Salt Lake City United States Biorepository and Molecular Pathology, Huntsman Cancer Institute, University of Utah, Salt Lake City, United States.

**Keywords:** Warthin tumor, Carcinoma, mucoepidermoid, Immunohistochemistry, Salivary gland neoplasms

## Abstract

**Objective:**

This study aimed to analyze the clinicopathological and immunohistochemical profiles of unconventional Warthin tumors and mucoepidermoid carcinomas.

**Methods:**

Eleven low-grade salivary gland tumors with Warthin-like features were analyzed. Group A (n=6) showed a double-layered oncocytic epithelium in less than 50% of the epithelial cells, whereas Group B (n=5) lacked this feature. Additionally, five conventional Warthin tumors and ten conventional mucoepidermoid carcinomas were analyzed as reference groups. Immunohistochemical staining was performed for cytokeratins (types 5/6, 7, and 14), p63, mitochondrial antigens, and Ki-67. Expression patterns were compared between groups.

**Results:**

Group A exhibited an oncocytic epithelium arranged in a double-layered pattern, with mitochondrial antigens strongly staining the luminal oncocytic cells. Cytokeratin 7 expression in Warthin tumors was predominantly luminal. In contrast, Group B showed glandular and cystic structures with diffuse mitochondrial antigen and cytokeratin 7 positivity. Immunohistochemical analysis revealed significant differences, with Warthin tumors showing distinct double-layered epithelial structures, and mucoepidermoid carcinomas displaying diffuse staining patterns.

**Conclusion:**

Distinct immunohistochemical profiles, including mitochondrial antigen and cytokeratin 7 expression, along with morphological differences, support the classification of Group A as metaplastic Warthin tumors and Group B as Warthin-like variants of mucoepidermoid carcinoma. These findings highlight the importance of molecular and immunohistochemical analyses for accurate classification of these tumors.

## In Brief

Bonfitto et al. compared metaplastic Warthin tumors and mucoepidermoid carcinomas with lymphoid stroma. Distinct CK7 and mitochondrial patterns, along with key morphological differences, support their distinction. These findings highlight the importance of integrating immunohistochemistry with molecular analyses for accurate classification.

## Highlights

■ Immunohistochemistry distinguishes Warthin-like mucoepidermoid carcinoma from metaplastic Warthin tumor.
■ CK7, mitochondrial patterns, and double-layer epithelium suggest metaplastic Warthin tumor.
■ Basal CK14, CK5/6, and p63 distinguish metaplastic Warthin tumor from Warthin-like mucoepidermoid carcinoma.

## Graphical abstract

**Figure:**
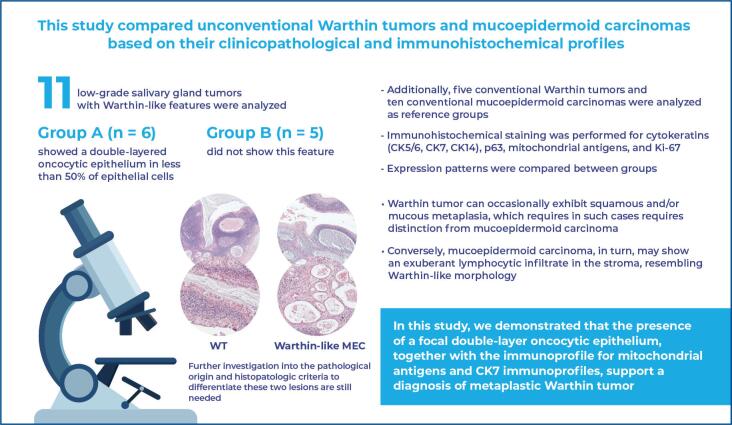

## INTRODUCTION

The morphological similarities and diagnostic challenges between Warthin tumor (WT) with metaplasia and mucoepidermoid carcinoma (MEC) with lymphoid stroma have recently been discussed.^(1–3)^ Warthin tumor is the second most common benign salivary gland tumor,^(4)^ whereas MEC is the most prevalent malignant neoplasm.^(5)^ Typically, WT is characterized by abundant lymphoid stroma, cysts lined by a double-layered oncocytic epithelium, and occasionally a papillary architectural pattern.^(6)^ Conversely, MEC often shows a prominent cystic component, epithelium with variable proportions of squamous, intermediate, and mucous cells, desmoplastic stroma, and a non-exuberant lymphoid infiltrate.^(7,8)^ However, WT can occasionally exhibit squamous and/or mucous metaplasia, making differentiation from MEC challenging.^(9,10)^ Additionally, MEC may present with a substantial lymphocytic infiltrate in the stroma, termed Warthin-like MEC.^(1,3,11–13)^

Currently, there is no clear histological definition for distinguishing these two types of tumors.^(1–3)^ Establishing histopathological criteria is crucial due to the clinical implications of misdiagnosing benign (*i.e.*, WT) and malignant (*i.e.*, Warthin-like MEC) tumors.

## OBJECTIVE

To analyze the clinicopathological and immunophenotypic characteristics of a series of morphologically unconventional Warthin tumors and mucoepidermoid carcinomas.

## METHODS

This study was approved by the Research Ethics Committee of the *Universidade Estadual de Campinas* (CAAE: 11521019.0.0000.5404; # 3.357.193). All procedures involving human participants were conducted in strict accordance with the ethical standards of the institutional research committee and the principles outlined in the Declaration of Helsinki (1964) and its subsequent amendments. Informed consent was obtained from all the participants. Consent was obtained in written form, ensuring that patient anonymity was rigorously maintained throughout the manuscript and publication process.

All cases were retrieved from the archives of the Department of Pathological Anatomy of the *Escola de Ciências Médicas* of the *Universidade Estadual de Campinas* and the Clinical Hospital of the same university to ensure consistency in sample processing and diagnostic evaluation.

### Study group selection

This study analyzed 11 low-grade epithelial salivary gland tumors characterized by glandular and/or cystic structures surrounded by abundant lymphoid tissue (Warthin-like pattern). The tumors were divided into two groups based on their histological features:

Group A (n=6): Tumors showing a double-layered oncocytic epithelium, similar to that observed in conventional Warthin tumors, but accounting for less than 50% of the total epithelial cells.

Group B (n=5): Tumors lacking the double-layered oncocytic epithelium, while still exhibiting glandular and/or cystic structures within abundant lymphoid stroma.

### Control Group selection

Two Control Groups were included for comparison with the unconventional tumors:

Conventional WT Control Group (n=5): Tumors showing a high frequency of double-layered oncocytic epithelium in more than 50% of the total epithelial cells.

Conventional MEC Control Group (n=10): Tumors lacking prominent peritumoral lymphoid stroma and exhibiting only the typical histological features of MEC.

All cases were classified according to the criteria established in the 2023 World Health Organization Classification of Head and Neck Tumors.^(5)^

### Data collection, histological evaluation and immunohistochemical analyses

Clinical data were collected, and Hematoxylin and Eosin (H&E)-stained histological slides were examined. Samples were grouped and semi-quantitatively analyzed on a scale from 0 to ++++, representing the absence or presence of morphological findings at different frequencies: 5 to 25% (+), 25% to 50% (++), 50% to 75% (+++), and more than 75% (++++) of the tumor area. Various morphological variables were evaluated, including the double-layered oncocytic epithelium, tumor-associated fibrosis, intermediate cell epithelium, squamous differentiation areas, mucous cells, cells with apocrine secretion, ciliated cells, and lymphoepithelium.

Immunohistochemical analysis was performed on 3-μm-thick paraffin sections of representative blocks, following the method of de Angelis et al.^(14)^ Primary antibodies included anti-p63 (DAKO, Clone 4A4, Dilution 1:500), anti-CK5/6 (DAKO, Clone D5/16B4, Dilution 1:100), anti-CK7 (DAKO, Clone OR-TL12/30, Dilution 1:100), anti-CK14 (DAKO, Clone LL002, Dilution 1:400), anti-mitochondria (Biogenex, Clone 113-1, Dilution 1:100) and Ki-67 (DAKO, Clone MIB-1, Dilution 1:100). Stained sections were semi-quantitatively evaluated, with immunoreactivity graded as follows: (0) no signal; (+) focal or <50% positive cells; and (++) diffuse or >50% positive cells. Semi-quantitative assessments of both morphological and immunohistochemical features were performed independently by three investigators to reduce subjective bias. Cases with discrepant interpretations were documented and included in the analyses.

## RESULTS

### Clinicopathological features of Groups A and B

**Figure 1:**
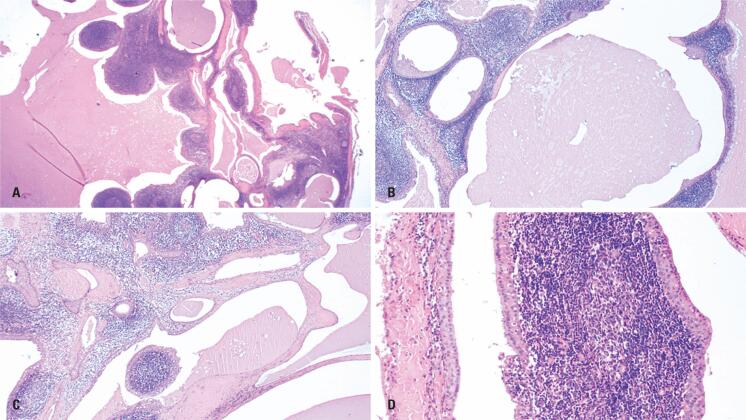
Histopathological features of Group A – Case 2. (A-C) Panoramic view showing a multicystic lesion with frequent papillary projections and associated lymphoid stroma, resembling a Warthin-like pattern (H&E, ×20, ×40, and ×40, respectively). (D) Focal areas display the characteristic double-layered oncocytic epithelium seen in conventional WT (H&E, ×100)

**Figure 2:**
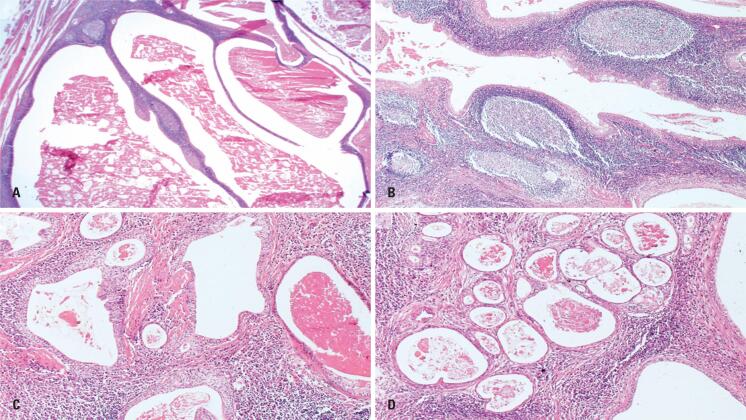
Histopathological features of Group B – Case 8. (A, B) Multicystic Warthin-like lesions with occasional small papillary projections and prominent lymphoid stroma (H&E, ×20 and ×40). (C, D) Glandular structures and microcysts embedded in fibrotic stroma and absence of the double-layered oncocytic epithelium characteristic of conventional WT (H&E, ×40 and ×100)

**Figure 3:**
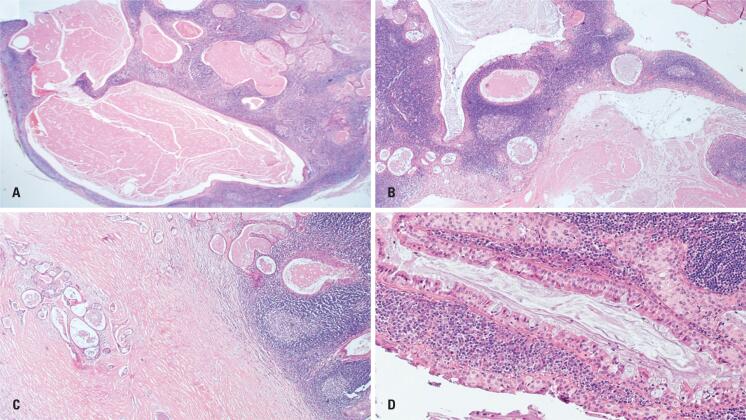
Histopathological features of Group B – Case 9. (A) Panoramic view showing a multicystic tumor with prominent lymphoid stroma (H&E, ×20). (B, C) Glandular structures and focal angulated microcysts within dense fibrotic bands (H&E, ×40). (D) Tumor epithelium displaying oncocytic, intermediate, and mucous cells, without definitive evidence of the double-layered oncocytic epithelium typical of conventional WT (H&E, ×100)

Detailed clinical and morphological features are provided in table 1. Semiquantitative histopathological assessment showed an interobserver concordance of 70%, whereas immunohistochemical evaluation showed a higher concordance rate of 98.3%. In Group A (Figure 1), a focal double-layered oncocytic epithelium was observed in less than half of the total epithelial cell population. Mucous and intermediate cells, along with tumor-associated fibrosis, were variably present, primarily at small focal points. Squamous differentiation was noted in one case, comprising over 50% of the tissue. Variable proportions of ciliated and apocrine cells, as well as lymphoepithelium, were also observed. Group B (Figures 2 and 3) did not show the typical double-layered oncocytic epithelium but had relatively common findings of mucous and intermediate cells, along with tumor-associated fibrosis. The squamous epithelium was infrequent compared to that in Group A. None of the cases displayed a significant frequency (>5% of the total tumor area) of ciliated and apocrine cells. Lymphoepithelial areas were observed in only two cases (40%) at focal points.

**Table 1:** Clinicopathological characteristics and frequency of histopathological features in parotid gland tumors of Groups A (n=6) and B (n=5)

#/Groups	Age/ Sex	Duration of symptoms (months)	Tumor size (cm)	Diagnosis	Double-layered oncocytic epithelium	Tumor-associated fibrosis	Squamous epithelium	Mucous cells	Intermediate Cells	Ciliated cells	Apocrine secretion	Lymphoepithelial areas
1/A	65/F	12	NI	WT	++	+	+ / 0	0 / +	+	0	+	+ / ++
2/A	61/M	5	4	WT	+	++	+++	+ / ++	++ / +	+ / ++	+ / 0	+
3/A	64/M	10	3.5	WT	+	+	+ / 0	++ / +++	+ / +++	++	0 / +	+
4/A	58/M	144	4	WT	++	+++ / ++	+ / 0	+	+	0	++ / +	+ / 0
5/A	50/M	12	4.5	WT	++	+	0	+	++	0	+	++
6/A	NI	NI	NI	NI	++	+	+	+	++	0	++	+
7/B	78/F	60	2.1	MEC	0	+++ / ++++	0	+++ / ++++	++ / +	0	0	0
8/B	65/F	NI	NI	WT	0	+++	0	+ / ++	+++	0	0	0 / ++
9/B	53/M	NI	NI	NI	0	++	+ / 0	++ / +++	++++ / ++	0	0	0 / +
10/B	13/M	5	NI	MEC	0	++++	0	+++	+	0	0	0
11/B	49/M	24	0.5	MEC	0	++++	++	++	+	0	0	0

The data were assessed by H&E staining using a semi-quantitative scoring system (two values separated by a slash (/) indicate discrepant interpretations).

Cases with not informed (NI) diagnosis or data were grouped according to their morphological features. The same applies to the single Warthin tumor case in Group B.

F: female; M: male; WT: Warthin tumor; MEC: mucoepidermoid carcinoma; NI: not informed.

### Cytokeratin immunohistochemical expression

**Figure 4:**
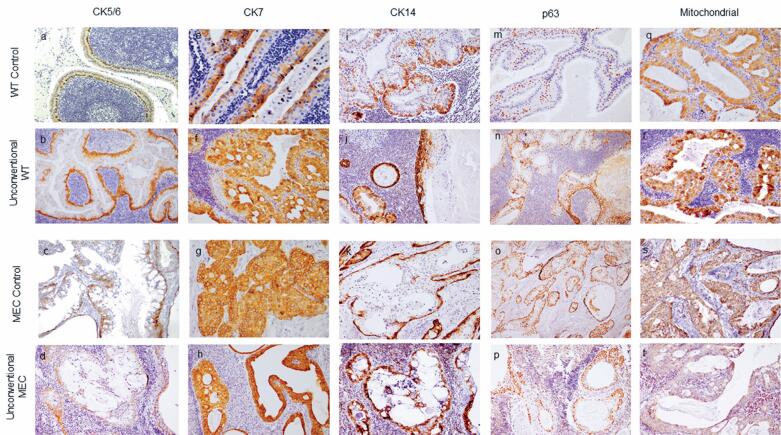
Immunohistochemical expression of CK5/6, CK7, CK14, p63, and mitochondrial antigens in conventional MEC and Warthin tumor (WT) controls, and in tumors from Groups A and B. (A-D) CK5/6 positivity is observed in tumors with higher proportions of intermediate and squamous cells, particularly prominent in MEC controls. (E-H) CK7 shows predominant expression in luminal columnar/oncocytic cells in Group A and WT controls, while diffuse expression across all cell types is seen in Group B and MEC controls. (I-L) CK14 identifies focal linear basal cells, even in Group A cases lacking characteristic double-layered oncocytic epithelium. (M-P) p63 immunoreactivity is primarily observed in the basal layer, with increased expression in intermediate cells across Group A, Group B, and MEC controls. (Q-T) Mitochondrial antigen staining indicates uniform moderate-intensity positivity throughout epithelial layers in WT groups, with distinct luminal cell positivity in one case from Group B

CK5/6 was predominantly expressed in the basal portions of the tumor nests and intermediate cell areas in the MEC controls. In the WT controls, CK5/6 expression was primarily linear in basal cells. Groups A and B exhibited immunoprofiles similar to those of their conventional counterparts, with the latter showing a higher number of positive intermediate cells (Figure 4A-D, Table 2).

**Table 2:** Predominant positivity of CK5/6, CK7, CK14, p63, and mitochondrial antigens in Groups A and B and control tumors

	CK5/6			CK7			CK14			p63			MIT		
	B	I/S	O/C	B	I/S	O/C	B	I/S	O/C	B	I/S	O/C	B	I/S	O/C
Group A	5/5 ++	1/5 +	5/5 0	6/6 0	6/6 0	6/6 ++	4/4 ++	4/5 +	5/5 0	6/6 ++	6/6 ++	6/6 0	6/6 +	6/6 +	6/6 ++
Group B	3/3 ++	3/3 0	3/3 0	4/4 ++	4/4 ++	4/4 ++	1/3 ++	2/3 +	3/3 0	4/4 ++	4/4 ++	4/4 0	5/5 ++	5/5 ++	3/5 ++
WT Control	4/4 ++	1/4 +	4/4 0	5/5 0	1/5 +	5/5 ++	4/4 ++/+	1/4 +	4/4 0	5/5 ++	1/5 ++	5/5 0	1/5 ++	1/5 ++	5/5 ++
MEC Control	7/9 ++	5/9 +	9/9 0	5/8 ++	8/8 ++	6/8 ++	8/10 ++	7/10 +	10/10 0	9/9 ++	9/9 ++	9/9 0	10/10 ++	10/10 ++	10/10 0

WT: Warthin tumor; MEC: mucoepidermoid carcinoma; B: basal cells; I/S: intermediate/squamous cells; O/C: oncocytic/columnar cells.

CK7 was predominantly present in the luminal oncocytic/columnar cells of WT controls, in contrast to MEC-control tumors, which exhibited diffuse positivity throughout the neoplastic epithelium. Similarly, Group A demonstrated a predominantly luminal antigen disposition, whereas Group B showed a larger number of positive cells without a characteristic pattern in the tumor glands (Figure 4E-H, Table 2).

CK14 was frequently observed in the basal portions of tumors in the controls and was distinctly expressed in a single basal cell layer in the WT controls. In Group A, CK14 was focally present in small areas, consistent with double-layer oncocytic epithelium. Group B had fewer positive basal cells than Group A (Figure 4I-L, Table 2).

### p63 immunohistochemical expression

p63 positivity was observed in basal cells of the WT controls, with a distinct pattern present in intermediate cells in only one tumor. In the MEC controls, Groups A and B had similar immunoprofiles, featuring frequent positive intermediate cells and continuous basal expression (Figure 4M-P, Table 2).

### Mitochondrial antigens immunohistochemical expression

The WT controls predominantly displayed high-intensity luminal positivity in columnar/oncocytic cells. In contrast, the MEC control specimens exhibited diffuse expression with lower intensity. All cases in Group A showed high-intensity antigen expression in luminal cells, whereas most cases in Group B were diffusely positive in the epithelial layers (Figure 4Q-T, Table 2).

### Ki-67 index

The Ki-67 proliferation index in control tumor epithelial cells was ≤5%, except for two MEC specimens, which scored 20% and 60%. In Group A, the Ki-67 index varied: one case had a score of 5%, three cases 10%, and two cases 25% and 30%, mainly in regions with intermediate and mucous cells and predominantly in the basal layers. None of the cases in Group B had a proliferation index score exceeding 5%.

## DISCUSSION

In this study, we conducted a comparative analysis of 11 low-grade epithelial salivary gland tumors with Warthin-like features. Group A comprised cases showing a double-layered oncocytic epithelium involving less than 50% of the total epithelial cells, whereas Group B included tumors lacking this morphological feature but exhibiting glandular and/or cystic structures within abundant lymphoid stroma. For reference, we compared these tumors with cases of conventional WT and MEC.

The primary criterion for inclusion in Group A was the presence of a double-layer oncocytic epithelium. However, upon closer examination, we identified other distinguishing features that were more prevalent in Group A than in Group B. These included ciliated and apocrine cells as well as the lymphoepithelium. Tumor-associated fibrosis was more commonly observed in Group B, indicating a heightened stromal response to dysregulated epithelial growth. The presence of intermediate cells (with and without squamous differentiation) and mucous cells varied in both groups, but was more pronounced in cases lacking the double-layer oncocytic epithelium.

Our study revealed significant similarities in the immunohistochemical positivity patterns, particularly for CK7 and mitochondrial antigens. In Group A and WT controls, CK7 was predominantly expressed in luminal columnar/oncocytic cells, a trend that was also observed with anti-mitochondrial antibodies. The mitochondrial antigen demonstrated a robust response in the luminal cells, which was distinct from the patterns observed in the basal and intermediate cells. In contrast, Group B and MEC controls exhibited diffuse CK7 expression across all cell types. Mitochondrial antigen staining indicated predominantly uniform moderate-intensity positivity throughout the epithelial layers, with one case in Group B showing focal yet distinct luminal cell positivity.

The basal cell markers CK14 and CK5/6 demonstrated consistent immunoprofiles across all four groups, with expression in the peripheral cells within the tumor nodules and glandular structures. CK14 was particularly valuable for identifying focal linear basal cells, even in Group A cases lacking the characteristic double-layered oncocytic epithelium. CK5/6 exhibited positivity in tumors with higher proportions of intermediate and squamous cells, a pattern that was particularly pronounced in MEC controls. p63 immunoreactivity was similar across Groups A and B and MEC controls, and was primarily observed in the basal layer, with increased expression in the intermediate cells. According to García et al.,^(1)^ anti-p63 positivity favors classification of the lesion as MEC. These observations can be attributed to a distinct immunoprofile, revealing a notable abundance of intermediate cells beneath the columnar and mucous cells, which aligns with our findings. Interestingly, the WT controls exhibited contrasting basal cell expression patterns, with most tumors displaying fewer intermediate cells.

Notable morphological and immunohistochemical parallels were observed between Group A and WT controls, including the presence of a double-layer oncocytic epithelium and an immunophenotype characterized by CK7, CK14, and mitochondrial antigens. One case from Group B showed distinct luminal positivity with anti-mitochondrial antibody staining, suggesting a closer resemblance to Group A and WT controls. However, most cases in Group B and MEC controls exhibited similar immunohistochemical profiles for CK7 and mitochondrial antigens.

The morphological and immunohistochemical findings of this study support the classification of Group A as metaplastic WT. Conversely, the absence of the typical double-layered epithelium and the frequent presence of desmoplastic stroma in Group B suggest that these should be classified as Warthin-like variants of MEC. Although Warthin-like MEC may present with a double-layered epithelium, this feature is often atypical. The subjectivity of visual assessment complicates the diagnosis when based solely on morphological criteria, especially in limited samples.^(15,16)^

In this context, molecular analysis, particularly the detection of *MAML2* gene rearrangements, becomes essential. The chromosomal translocation t(11;19)(q21;p13), responsible for the *CRTC1-MAML2* fusion, is present in 38–82% of MEC cases and serves as a more precise diagnostic marker. Fluorescence in situ hybridization (FISH) is the recommended method for identifying this fusion and confirming MEC diagnosis.^(15,17)^ Lei et al.^(13)^ reported two cases initially diagnosed as metaplastic WT that were reclassified as Warthin-like MEC after detection of *MAML2* gene rearrangements by FISH. These findings emphasize the need for a combined diagnostic approach to avoid errors in cases with challenging morphological features.

Immunohistochemistry is an important complementary tool, particularly when molecular analyses are unavailable. Previous studies have highlighted the importance of markers such as CK5/6, CK14, and p63 in differentiating salivary tumors such as WT and MEC. However, our findings suggest that the inclusion of CK7 and mitochondrial antigens may expand the diagnostic options, particularly in morphologically atypical cases. Yang et al.^(18)^ observed that the eosinophilic double-layered epithelium was absent in 73.5% of Warthin type MEC cases, making morphological diagnosis difficult. In the remaining cases, the epithelium was flattened and less oncocytic. While immunohistochemistry with markers, such as p40, p63, CK5/6, CK7, and CA125, is useful for differentiation, the analysis of genetic rearrangements, particularly the *CRTC1-MAML2* fusion identified by FISH, is crucial for the definitive and accurate diagnosis of MEC.^(13,15,17)^

This study has several limitations. Molecular analyses targeting *MAML2* rearrangements were not performed in all cases. To ensure the accuracy of the diagnostic classification of WT and MEC, it is crucial to perform molecular analyses to confirm the presence or absence of *MAML2* rearrangements in both tumor groups. This approach would provide a more robust foundation for distinguishing these lesions and complement the histological and immunohistochemical findings. Moreover, while the results with CK7 markers and mitochondrial antigens are promising, the limited number of cases analyzed may affect their reliability. Validation of these markers in a larger sample size is necessary to confirm their specificity and sensitivityand to strengthen the conclusions. Expanding the sample and incorporating molecular testing would enable a more comprehensive and reliable diagnostic approach for these lesions.

Despite these limitations, our findings have important implications for clinical practice. Differential diagnosis between WT and MEC can be challenging, especially in atypical cases. Distinct immunohistochemical patterns, such as CK7 expression and mitochondrial antigens in WT, provide valuable diagnostic clues that may assist pathologists even in the absence of molecular tests. Accurate differentiation is essential as it directly affects treatment decisions, which varies depending on whether the lesion is benign or malignant. These results provide a basis for future investigations exploring the specificity and sensitivity of these markers to improve diagnostic accuracy.

## CONCLUSION

Our results indicate that CK7 and mitochondrial markers are useful for differentiating Warthin tumor from mucoepidermoid carcinoma, particularly based on their distinct expression patterns in luminal cells. However, validation using larger sample sizes is needed to confirm their specificity and sensitivity. The analysis of *MAML2* rearrangements is crucial for accurate molecular diagnosis and to define the appropriate treatment. The combination of established basal markers, such as CK5/6, CK14, and p63, with newer indicators, such as CK7 and mitochondrial markers, represents a step forward in improved diagnosis. Further studies with larger samples and more detailed molecular analyses are needed to consolidate these findings.

## Data Availability

After publication, data will be available from the authors upon request—this condition is justified in the manuscript.
